# CUI-Curate: a GraphRAG-based framework for automated clinical concept curation for NLP applications

**DOI:** 10.1093/jamia/ocag110

**Published:** 2026-06-26

**Authors:** Victoria Blake, Jamie Novak, Mathew Miller, Sze-Yuan Ooi, Blanca Gallego

**Affiliations:** Centre for Big Data Research in Health, University of New South Wales, Randwick, NSW 2031, Australia; Eastern Heart Clinic, Prince of Wales Hospital, Randwick, NSW 2031, Australia; Department of Cardiology, Prince of Wales Hospital, South Eastern Sydney Local Health District, Randwick, NSW 2031, Australia; NSW Ambulance Aeromedical Operations, Bankstown Helicopter Base, Bankstown, NSW 2200, Australia; Department of Anaesthesia, Saint George Hospital, Kogarah, NSW 2217, Australia; School of Clinical Medicine, University of New South Wales, Kingsford, NSW 2033, Australia; Eastern Heart Clinic, Prince of Wales Hospital, Randwick, NSW 2031, Australia; Department of Cardiology, Prince of Wales Hospital, South Eastern Sydney Local Health District, Randwick, NSW 2031, Australia; School of Clinical Medicine, University of New South Wales, Kingsford, NSW 2033, Australia; Centre for Big Data Research in Health, University of New South Wales, Randwick, NSW 2031, Australia

**Keywords:** clinical natural language processing, Unified Medical Language System, knowledge bases, large language models, phenotype

## Abstract

**Background:**

Clinical named entity recognition tools commonly map free text to Unified Medical Language System (UMLS) Concept Unique Identifiers (CUIs). For many downstream tasks, however, the clinically meaningful unit is not a single CUI but a *concept set* comprising related synonyms, subtypes, and associated concepts. Constructing these sets is labour-intensive, inconsistently performed, and poorly supported by existing tools.

**Methods:**

We present CUI-Curate, a graph-based retrieval-augmented-generation (GraphRAG) framework for automated UMLS concept set curation. A UMLS knowledge graph was constructed and embedded for semantic retrieval. Candidate CUIs were retrieved using graph-based expansion and then filtered and classified using large language models (GPT-5 and Qwen3-32B). The framework was evaluated on five lexically heterogeneous clinical concepts against manually curated concept sets and gold-standard concept sets.

**Results:**

CUI-Curate produced substantially larger and more complete concept sets than the manual benchmarks. A single retrieval configuration across concepts achieved high recall of definitive concepts with manageable candidate sets. GPT-5 outperformed manual curation for all concepts and retained at least 95% of definitive gold-standard CUIs, while Qwen3-32B achieved comparable but slightly lower performance. Many missed concepts were not observed in 10,000 MIMIC-III notes. CUI-Curate infrastructure and end-to-end processing were inexpensive and stable across runs.

**Conclusions:**

CUI-Curate offers a scalable, reproducible, and cost-efficient approach for generating clinician-reviewable UMLS concept sets tailored to clinical natural language processing and phenotyping applications.

## Introduction

Clinical natural language processing (NLP) tools often map clinical terms to Concept Unique Identifiers (CUIs) from the Unified Medical Language System (UMLS).^1^ The UMLS contains more than four million biomedical concepts drawn from over 180 source vocabularies, giving broad coverage of the many ways clinicians describe conditions, findings, and interventions, making it a powerful foundation for clinical concept normalization.^1^^,^^2^ However, its breadth and inclusivity also create challenges as concepts can overlap, use ambiguous wording, or be linked inconsistently through parent-child or synonymous relationships.^1^ As a result, it can be difficult to reliably identify all textual expressions that indicate a given clinical concept when extracting information from text.

For many downstream tasks, the clinically meaningful unit is not a single concept, but a set of related concepts encompassing synonyms, subtypes, and supertypes. For example, identifying conditions such as *heart failure* requires grouping individual CUIs such as *chronic heart failure*, *congestive heart failure* or *left-sided heart failure* into a list of related concepts that together represent a single clinical idea. These lists, often referred to as concept sets or value sets, define which CUIs should be treated as evidence for the target concept and are a key process if concepts from text are to be comparable and reproducible across datasets.^3^ This is a particular challenge in NLP applications, where concept normalization tools may map the same clinical expression to different CUIs depending on context, wording, or tool behavior as illustrated in Figure 1.

**Figure 1.**
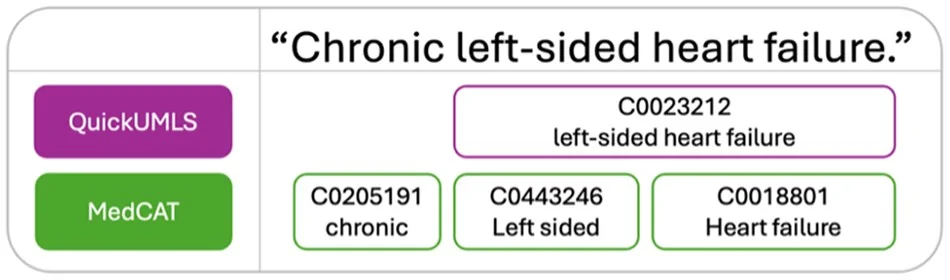
Example variation in concept normalization across NLP tools. The same clinical phrase is mapped to different CUIs by the QuickUMLS^4^ and MedCAT^5^ tools due to differences in tool behavior.

Constructing such concept sets is a manual and labor-intensive process.^6^ It requires specialist clinical judgment, iterative searching, and careful review to decide which concepts are relevant and which should be included. Existing tools offer only partial support. The UMLS Metathesaurus Browser^7^ enables manual exploration of CUIs, but offers limited support for collecting groups of related concepts as a coherent concept set. The Observational Health Data Sciences and Informatics (OHDSI) Athena^8^ platform is purpose built for constructing concept sets and provides more advanced search and hierarchical expansion across multiple vocabularies, but still relies heavily on manual curation. Moreover, Athena concept sets are designed primarily for structured, coded data and do not operate directly on UMLS CUIs, requiring additional mapping steps when used in NLP pipelines that extract concepts from free text. As a result, concept sets remain time-consuming to build and difficult to maintain.

Some vocabularies within the UMLS, such as Systematized Nomenclature of Medicine - Clinical Terms (SNOMED CT), provide hierarchical relationships that can be used to identify related concepts. While these hierarchies are useful, relying on hierarchical expansion alone is insufficient for constructing clinically meaningful concept sets. Relevant variants may appear at different levels of the hierarchy or outside a single branch, while broader parent concepts can introduce clinically inappropriate terms. Also, clinically relevant variants are not always directly connected through parent-child relationships, reflecting differences in how concepts are organized across source vocabularies. Figure 2 illustrates how CUIs relevant to a single clinical concept may be distributed across the UMLS graph, highlighting the limitations of hierarchy-based retrieval.

**Figure 2.**
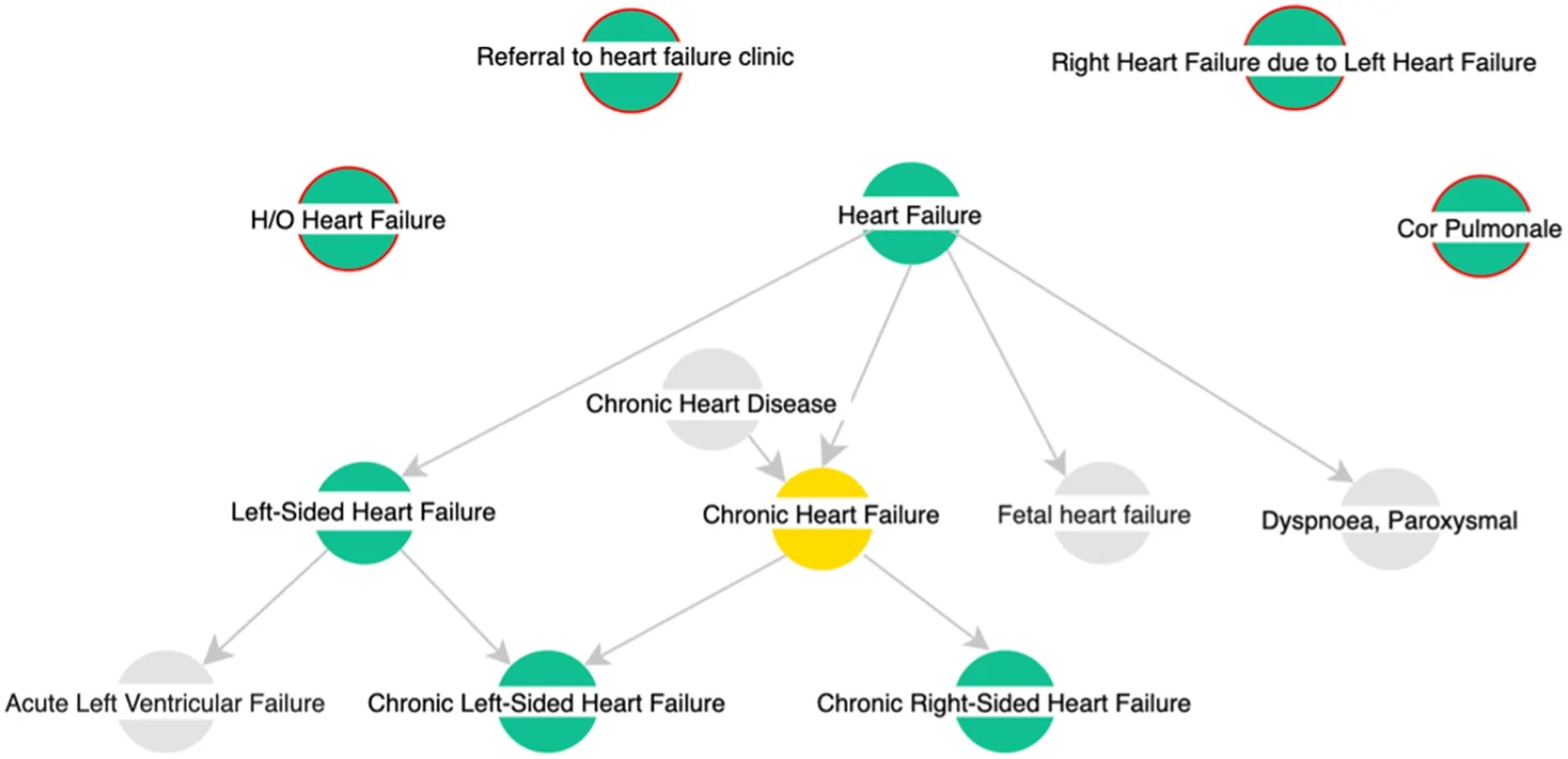
Example UMLS subgraph centered on chronic heart failure. The target concept is shown in yellow, clinically relevant variants in green, and related but clinically inappropriate concepts in grey. Nodes outlined in red represent relevant CUIs that are not directly connected to the target concept and would be missed by hierarchy-based retrieval. Arrows represent hierarchical relationships (parent to child) encoded in the UMLS.

The automation of concept set construction has received little direct attention. Large language models (LLMs) have demonstrated strong performance on a range of biomedical reasoning tasks, including assessing semantic relevance and handling clinical nuance,^9^ making them well suited to judging whether concepts are meaningfully related even when they are not hierarchically connected. However, applying LLMs directly to the full UMLS is impractical due to its size, cost, and the risk of overwhelming the model with irrelevant information. Moreover, approaches that rely on flat lexical or embedding-based retrieval alone ignore the rich relational structure of biomedical terminologies. As a result, LLMs require a constrained and clinically relevant search space in order to be applied effectively and efficiently to concept curation tasks.

Retrieval-augmented generation (RAG)^10^ provides a mechanism for constraining LLM inputs by retrieving a subset of relevant items to include as context. Graph-based variants of RAG known as GraphRAG^11^ extend this idea by performing retrieval over a graph structure rather than a flat document collection, enabling both semantic similarity and explicit relationships to be used during retrieval. This approach is particularly well suited to knowledge sources such as the UMLS, which is inherently graph-structured and encodes relationships between concepts across multiple vocabularies. In this study, we propose CUI-Curate, a GraphRAG-based framework that combines graph-based retrieval over the UMLS with LLM-based filtering and classification to automate the construction of clinically meaningful concept sets. By first retrieving a focused subgraph of candidate CUIs and then applying LLM reasoning to assess relevance and contextual certainty, this approach aims to reduce manual effort while producing curated clinically coherent concept sets that can be rapidly reviewed by clinicians and applied in NLP pipelines that map text to UMLS CUIs.

### Related work

Several initiatives have standardized clinical concepts through curated codes lists, including OHDSI concept sets and the National Library of Medicine’s Value Set Authority Center (VSAC).^3^ These resources support reproducible analysis of structured data but are typically tailored to specific studies and built around terminologies such as SNOMED CT or International Classification of Diseases (ICD), limiting direct applicability to NLP pipelines based on UMLS CUIs.^3^^,^^12^

Prior work on phenotype concept set construction includes supervised approaches that learn from existing curated phenotypes.^6^ However, these methods rely on prior phenotype definitions and do not target UMLS-based NLP workflows. Large language models have also been applied to concept normalization, where detected text mentions are mapped to the most appropriate UMLS concept. Dobbins et al. improved mention-level normalization by combining existing tools with LLM-based synonym generation and candidate pruning.^13^ Graph-based retrieval has similarly been explored in biomedical question answering and reasoning tasks, where GraphRAG provides structured context for LLM reasoning.^14^^,^^15^

However, the construction of reusable UMLS concept sets for downstream NLP applications remains largely manual and disconnected from concept extraction pipelines.

## Methods

### Data sources

The UMLS Rich Release Format (RRF) files were used to construct the knowledge graph, including concept names (MRCONSO), relationships (MRREL), definitions (MRDEF), and semantic types (MRSTY). Three source vocabularies with well-defined relational structure were included: SNOMED CT–US, the NCI Thesaurus (NCIt), and Medical Subject Headings (MeSH).

### Target concepts

Five lexically heterogeneous concepts representing a diverse range of clinical conditions and also known to have extensive subtypes or varied ways of being expressed were selected to develop and test CUI-Curate, which included *chronic heart failure (CHF), ischemic stroke (IS)*, *fluid overload (FO)*, *left ventricular systolic dysfunction (LVSD)* and *poor mobility (PM*).

### Knowledge graph construction, node embedding, and indexing

A bi-directed knowledge graph was constructed from UMLS relationships, with CUIs represented as nodes and semantic relationships encoded as edges. Node attributes included synonyms, definitions, and semantic types. Each node was embedded using three models representing general-purpose, domain-specific biomedical, and open-source general-purpose embeddings: *text-embedding-3-large* (OpenAI), a high-performing general-purpose closed model for semantic similarity and retrieval tasks,^16^  *SapBERT,* a BERT-based model trained using UMLS synonym pairs,^17^ and *e5-large-v2* an open-source general-purpose embedding model with strong performance across diverse embedding clinical and biomedical benchmarks.^18^^,^^19^ Embeddings were indexed using the FAISS cosine similarity algorithm (*IndexFlatIP)*.^20^ An overview of the CUI-Curate framework is shown in Figure 3.

**Figure 3.**
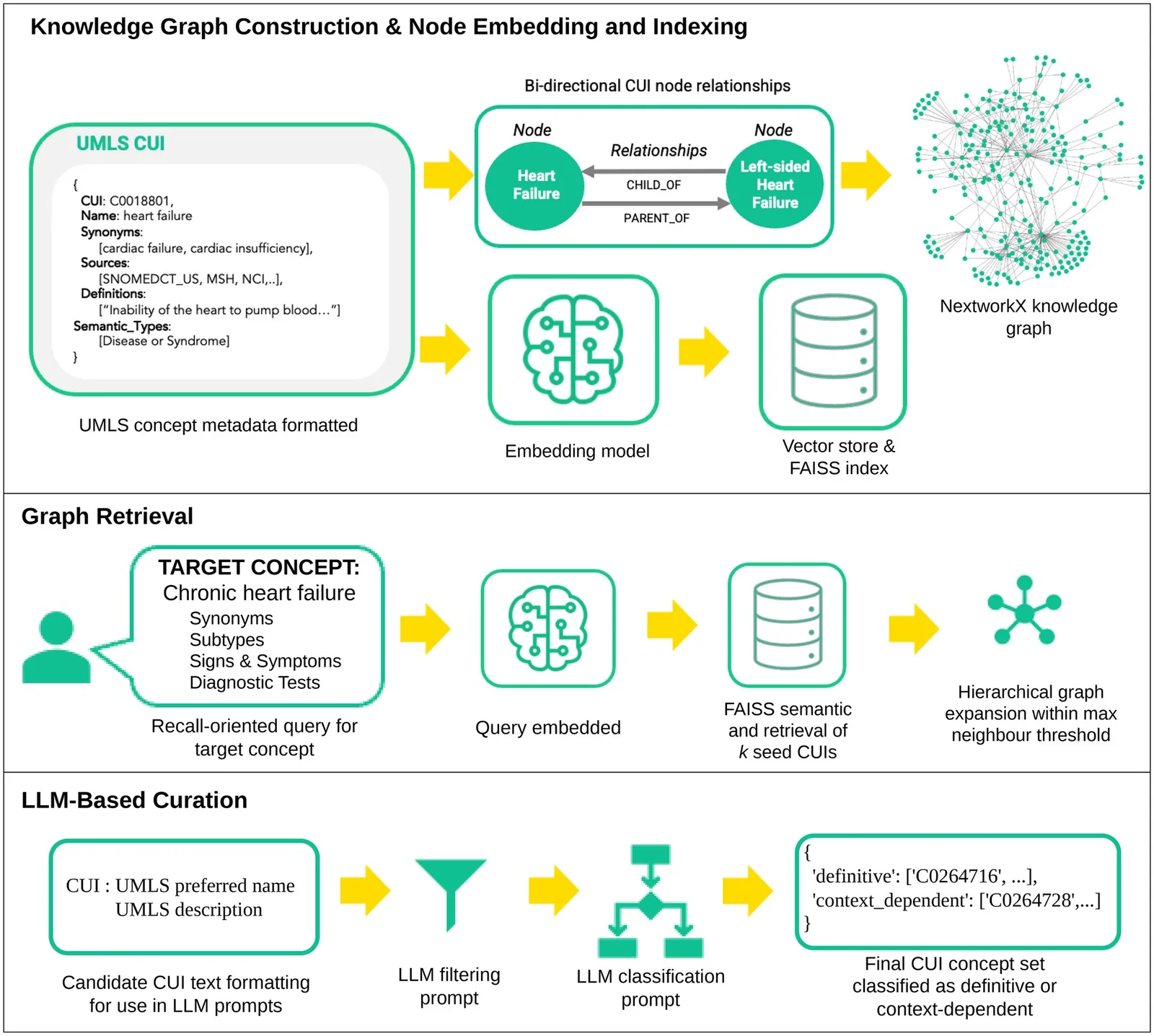
Overview of CUI-Curate, a GraphRAG-based framework for automated UMLS concept set curation. UMLS concepts were represented as a knowledge graph. Each concept forms one node within the graph with UMLS relationships forming the node edges. Each node was embedded using an embedding and indexed using FAISS model for semantic retrieval. For a given target concept, a recall-oriented description is embedded using the same embedding model which is used to query the FAISS index, retrieving k nearest-neighbor seed CUIs from the graph, which are expanded through hierarchical graph relationships to a threshold of semantic max neighbor CUIs, to form a focused candidate set. This CUI candidate set is then filtered for relevance and classified by an LLM chat model into definitive and context-dependent indicators of the target concept. Target concept descriptions can be found in the supplementary materials. Abbreviations: UMLS, Unified Medical Language System; CUI, Concept Unique Identifier; FAISS, Facebook AI Similarity Search; LLM, Large Language Model.

### Graph retrieval

Graph retrieval was designed to identify a high-recall but computationally manageable set of candidate CUIs for subsequent LLM curation. For each target concept, a recall-oriented description was embedded and used to retrieve the top-*k* semantically similar CUIs. Recursive child expansion along with one-hop parent and synonymous graph expansion (*hops*) incorporated hierarchical related concepts. Candidate sets were constrained using semantic type filters and neighborhood limits (*max_neighbors*) to maintain manageable prompt sizes. Additional details and parameter definitions are provided in the Supplementary materials.

### LLM-based curation

Retrieved CUIs were curated using a two-stage LLM workflow. First, CUIs were filtered to retain concepts that indicate or strongly suggest, the target phenotype. Second, included CUIs were classified as *definitive*, meaning explicitly or strongly indicating the target concept, or *context-dependent,* meaning the concept may indicate the target concept, but requires additional clinical information for confirmation.

Two chat models were compared: GPT-5 accessed through the OpenAI API, and Qwen3-32B,^21^ an open-source LLM that was deployed locally. A 4-bit quantized version of Qwen3-32B was used to enable inference on a single high-memory GPU. Both models were run five times for each target concept to assess variability in LLM outputs.

Prompt templates and implementation details are provided in the Supplementary materials.

### Benchmark and gold-standard concept sets

In the absence of existing benchmark concept sets for the selected target concepts, manual concept sets (*M*) were collected by two clinicians using the UMLS Metathesaurus Browser and adjudicated by a third clinician. Discrepancies were resolved by a third clinician, and agreement between the independently collected sets was assessed using Jaccard and overlap coefficients.

Because exhaustive review of all potentially relevant CUIs in the UMLS Metathesaurus was infeasible, an adjudicated gold-standard concept set (*G_a_*) was constructed using pooled relevance assessment,^22^ a standard methodology developed in information retrieval evaluation to create reference standards from the union of candidates retrieved by multiple systems. This approach has been widely used in Text REtrieval Conference (TREC) evaluations and has been shown to remain robust for evaluating modern neural retrieval systems.^23^

Candidate CUIs retrieved using the three embedding models described above, together with graph traversal using hierarchical and hop neighborhood expansion, were combined to form a comprehensive candidate retrieval pool that was independently annotated by two clinicians. Disagreements were resolved by consensus. Interrater agreement was assessed using percent agreement and Cohen’s κ. The resulting adjudicated set of included CUIs formed the gold-standard concept set (*G_a_*) used for downstream evaluation.

### Evaluation framework

Each stage of the pipeline was evaluated separately using concept sets accessible to that stage (ie, the method’s retrieval or inclusion space) to ensure fair comparisons. Retrieval was evaluated against the adjudicated gold-standard concept sets (*G_a_)*, using recall as the primary optimization target.

Filtering Performance for both the LLMs and the manual benchmark (*M*) was evaluated against the subset of *G_a_* available in each run’s retrieved candidate set using precision, recall, and F1-score. Preservation of definitive concepts was evaluated using the same metrics, with the reference standard restricted to the subset of *G_a_* CUIs classified as *definitive*.

Additional evaluation methodological details are provided in the supplementary materials.

### Clinical text observability analysis

To assess the practical significance of missed concepts, CUIs missed during retrieval and filtering were evaluated for occurrence in a sample of 10,000 MIMIC-III clinical notes sampled across multiple document types using QuickUMLS. A similarity threshold of 0.7, score-based overlap resolution, and a 10-token matching window was used. For each target concept, gold-standard CUIs missed at graph retrieval and after LLM filtering were classified as observed or not observed in the sampled notes. This analysis was intended as a pragmatic assessment of whether recall losses affected CUIs appearing in the sampled clinical text.

CUI-Curate is available as a freely available Python-based package which can be accessed through the CUI-Curate GitHub repository (https://github.com/vickyblake/CUICurate). Source code, prompt templates, and adjudicated gold-standard concept sets are publicly available in the repository.

## Results

### Knowledge graph

The UMLS graph constructed from SNOMED CT, MeSH, and NCIt vocabularies comprised 924,211 nodes and 1,429,114 edges. After restricting the graph to the semantic types used in the GraphRAG pipeline, the working subgraph comprised 144,646 nodes and 223,043 edges. In this restricted graph, the median node degree increased to 2 (IQR 1-3), and 8.2% of nodes were isolated.

### Manual and gold-standard concept sets

Manual concept sets (*M*) identified through the UMLS browser were substantially smaller than the pooled gold-standard sets (*G_a_*), ranging from 30-130 CUIs compared with 163-675 CUIs (Table 1). The largest *G_a_* sets were observed for IS and PM, reflecting the broad and heterogeneous ways in which these concepts can be represented in clinical terminology. CHF had the highest proportion of definitive concepts (69.6%), and fluid overload had the highest proportion of context-dependent concepts (92.8%). Agreement between clinicians on inclusion decisions within the pooled *G_a_* candidate sets was moderate to high, with percent agreement between 73.7% and 94.5% and Cohen’s κ from 0.35–0.87.

**Table 1.** Manual and adjudicated gold-standard concept set composition and annotator agreement statistics.

Source	Concept	Concept set classifications			Agreement statistics			
					Inclusion		Category	
		Total *n*	Definitive *n* (%)	Context-dep *n* (%)	IAA (%)	κ	IAA (%)	κ
**Manual concept sets (*M*)**	**CHF**	98	48 (49.0%)	50 (51.0%)				
	**FO**	30	15 (50.0%)	15 (50.0%)				
	**IS**	130	79 (60.8%)	51 (39.2%)				
	**LVSD**	55	23 (41.8%)	32 (58.2%)				
	**PM**	92	28 (30.4%)	64 (69.6%)				
**Gold-standard adjudicated concept sets (*G_a_*)**	**CHF**	163	114 (69.9%)	49 (30.1%)	82.3%	0.35	67.5%	0.31
	**FO**	321	23 (7.2%)	298 (92.8%)	90.2%	0.54	82.4%	0.52
	**IS**	675	403 (59.7%)	273 (40.4%)	93.9%	0.87	85.7%	0.66
	**LVSD**	287	51 (17.8%)	236 (82.2%)	94.5%	0.76	75.3%	0.47
	**PM**	655	355 (54.2%)	300 (45.8%)	73.7%	0.39	73.6%	0.43

Note: Manual concept sets (M) collected directly from the UMLS Metathesaurus browser. Adjudicated gold-standard (*G_a_*) sets constructed by adjudicating the pooled union of candidate CUIs retrieved using three embedding models and graph expansion strategies. Agreement statistics are only shown for the (*G_a_*) concept sets, for which both clinicians independently reviewed the same pooled candidate CUIs. Because the (M) concept sets were generated through independent open-ended UMLS browsing rather than assessment of a fixed candidate set, agreement was summarized using Jaccard and overlap coefficients which are provided in the supplementary materials.

Abbreviations: CHF, chronic heart failure; Context-dep, context-dependent; FO, fluid overload; IS, ischemic stroke; LVSD, left ventricular systolic dysfunction; PM, poor mobility.

### Graph retrieval performance

Retrieval performance varied substantially across target concepts (Figure 4). CHF showed the strongest retrieval performance, whereas FO and PM were more challenging to retrieve comprehensively. Across most settings, text-embedding-3-large outperformed SapBERT and e5-large-v2 although differences between models were smaller for the two disease concepts CHF and IS. Recall gains diminished for most concepts beyond approximately 500 seed CUIs. Definitive CUIs were generally retrieved with higher recall at smaller values of *k* and fewer retrieved CUIs, particularly for FO and LVSD, where the definitive subsets were relatively small (23 and 51 CUIs, respectively).

**Figure 4.**
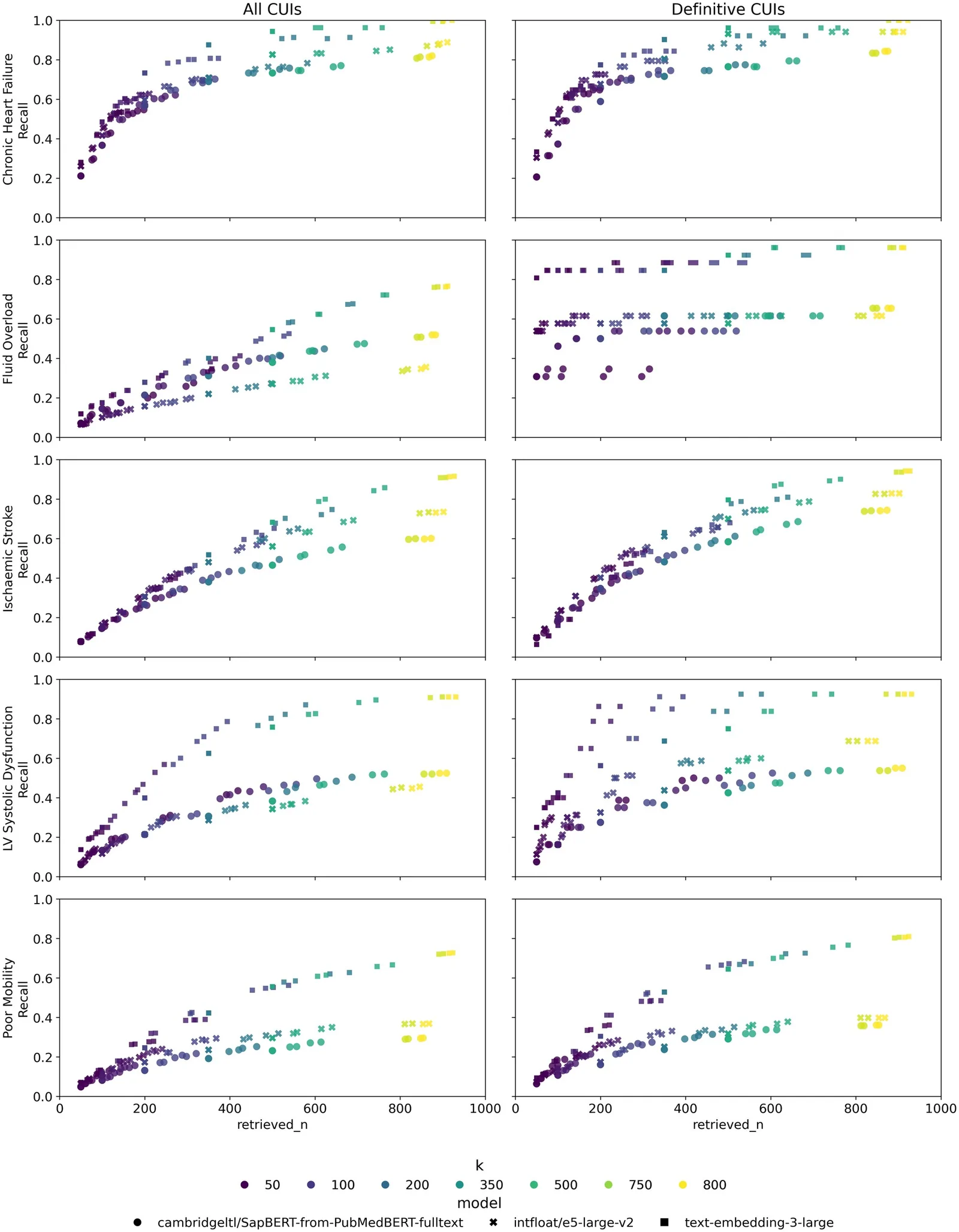
Retrieval performance across embedding models and graph retrieval settings. Scatter plots show recall of adjudicated gold-standard concept sets (left column, all CUIs; right column, definitive CUIs only) versus the number of CUIs retrieved for five target concepts. Each point represents a unique retrieval configuration varying the number of semantically retrieved seed CUIs (k = 50-800; color scale), embedding model (marker shape), plus 0 or 1 expansion hops and max neighbor thresholds (50–1,000). Across most concepts, text-embedding-3-large achieved the highest recall, and recall gains diminished beyond approximately 500 seed CUIs. Definitive CUIs were generally retrieved with higher recall than the full gold sets. Abbreviations: CUI, concept unique identifier; CHF, chronic heart failure; FO, fluid overload; IS, ischemic stroke; LVSD, left ventricular systolic dysfunction; PM, poor mobility.

To assess the feasibility of achieving complete recall, additional analyses were conducted using semantic-only retrieval and unrestrained (no max neighbors) semantic retrieval with recursive child expansion. However, these approaches often became computationally impractical for downstream LLM filtering, particularly for broad and semantically diffuse target concepts. Semantic-only retrieval alone required tens of thousands of CUIs to achieve complete recall, particularly for FO and PM (Supplementary Table). Unrestricted child expansion (Table 2) substantially improved recall but frequently produced large increases in candidate set size and required multiple levels of hierarchical traversal. Using 500 seed CUIs, FO recall increased from 0.55 to 0.83, but expanded the candidate set to 2,547 CUIs, while PM recall increased from 0.44 to 0.66 but with 2,056 retrieved CUIs.

**Table 2.** Semantic retrieval and unrestricted child expansion recall and number of retrieved CUIs across target concepts.

Concept	Metric	Seeds (k)					
		250	500	750	800	1000	1500
**CHF**	Seed recall	0.77	0.94	0.99	1.00	1.00	1.00
	Seed + child recall	0.83	0.96	0.99	1.00	1.00	1.00
	Min child depth	2	1	0	0	0	0
	CUIs retrieved	529	1040	750	800	1000	1500
**FO**	Seed recall	0.36	0.55	0.68	0.70	0.78	0.83
	Seed + child recall	0.76	0.83	0.89	0.89	0.90	0.93
	Min child depth	7	3	4	4	4	4
	CUIs retrieved	2472	2547	3981	4038	6129	7909
**IS**	Seed recall	0.33	0.60	0.79	0.81	0.89	0.95
	Seed + child recall	0.67	0.85	0.95	0.95	0.98	0.99
	Min child depth	5	4	4	4	4	3
	CUIs retrieved	1037	1469	1841	1915	2082	2812
**LVSD**	Seed recall	0.44	0.66	0.80	0.82	0.87	0.92
	Seed + child recall	0.70	0.87	0.92	0.93	0.94	0.96
	Min child depth	3	3	2	2	2	1
	CUIs retrieved	497	1314	1831	2706	3499	2926
**PM**	Seed recall	0.25	0.44	0.58	0.60	0.70	0.74
	Seed + child recall	0.53	0.66	0.76	0.77	0.79	0.82
	Min child depth	4	6	3	3	3	3
	CUIs retrieved	1129	2056	2344	2389	3319	4686

Note: For each target concept, the maximum achievable recall was calculated using the top-k semantically retrieved seed concept unique identifiers (CUIs) and unrestrained child expansion through child hierarchical relationships. Seed recall represents the recall achievable with the seeds alone, and seed + child recall represents the recall achieved through child expansion from those seeds. Min child depth represents the number of child traversals needed to reach the maximum seed + child recall. CUIs retrieved represents the number of candidate CUIs returned from this unbounded seed + child expansion.

Abbreviations: CHF, chronic heart failure; FO, fluid overload; IS, ischemic stroke; LVSD, left ventricular systolic dysfunction; PM, poor mobility.

Based on these analyses, a single operating point was selected using text-embedding-3-large, 500 seed CUIs, one-hop expansion, and a neighborhood limit of 1,000 generated approximately 750 candidate CUIs per concept while maintaining high recall (Table 3). Overall recall ranged from 0.66 to 0.96, and definitive recall exceeded 0.90 for all concepts except PM.

**Table 3.** Selected GraphRAG retrieval settings and retrieval performance at those settings across target concepts.

Concept	CUIs retrieved	Overall recall	Definitive recall
**CHF**	758	0.96	0.96
**FO**	768	0.72	0.96
**IS**	764	0.85	0.90
**LVSD**	743	0.89	0.92
**PM**	782	0.67	0.77

Note: The same retrieval settings were used across concepts which were text-embedding-3-large embeddings, k = 500 seed CUIs, max neighbors = 1000, and one-hop graph expansion. Overall recall was calculated against the full adjudicated gold-standard sets (*G_a_*), while definitive recall was calculated using only *G_a_* CUIs classified as definitive indicators of the target concept.

Abbreviations: CHF, chronic heart failure; CUI, concept unique identifier; FO, fluid overload; IS, ischemic stroke; LVSD, left ventricular systolic dysfunction; PM, poor mobility.

### Filtering performance

Figure 5 summarizes filtering performance. GPT-5 achieved the highest recall and F1-scores for both the full gold-standard concept sets (*G_a_*) and the subset of definitive CUIs. Qwen3-32B consistently outperformed *M* set construction in recall but generally underperformed GPT-5, particularly for FO and LVSD.

**Figure 5.**
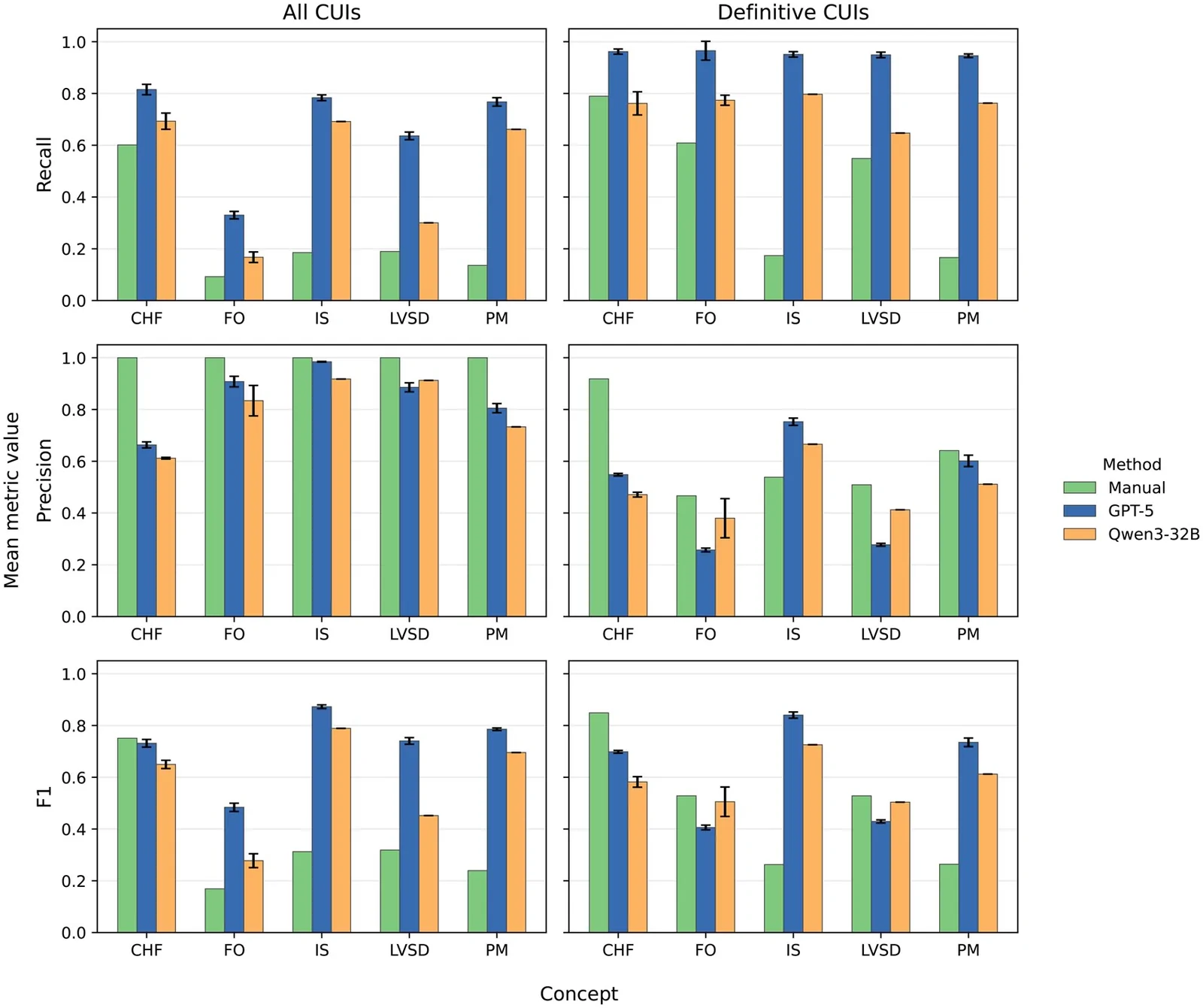
LLM filtering performance. Mean recall, precision, and F1-score across five runs per concept for GPT-5 and Qwen3-32B, compared with manual concept set collection (manual). Performance is shown for the full adjudicated gold-standard concept sets (left column, All CUIs) and for the subset of definitive CUIs only (right column, Definitive CUIs). Error bars represent the standard deviation in performance. LLM outputs evaluated against the adjudicated gold-standard concept sets using CUIs available in each method’s retrieved candidate set. GPT-5 achieved the highest recall and F1-scores across most concepts. Recall was substantially higher when evaluation was restricted to definitive CUIs with GPT-5 retaining at least 95% of definitive phenotype-defining concepts across the five target concepts. Abbreviations: CUI, concept unique identifier; CHF, chronic heart failure; FO, fluid overload; IS, ischemic stroke; LVSD, left ventricular systolic dysfunction; PM, poor mobility. Full performance metrics can be found in the Supplementary materials.

For the full *G_a_* concept sets, GPT-5 recall ranged from 0.33 for FO to 0.82 for CHF, with particularly strong performance for IS (0.78), PM (0.77) and LVSD (0.64) in comparison to *M* concept sets which achieved markedly lower recall for all concepts except CHF, ranging from 0.09 for FO to 0.60 for CHF. Qwen3-32B showed intermediate performance, with recall ranging from 0.17 to 0.69.

Recall was substantially higher when evaluation was restricted to definitive CUIs. GPT-5 achieved recall of 0.95 or greater for all five concepts, demonstrating near-complete retention of the most clinically specific concepts despite more modest recall for the full *G_a_* concept sets. Qwen3-32B also maintained substantially better recall for definitive CUIs than for the complete concept sets, ranging from 0.65 to 0.80. *M* sets showed substantially lower inclusion of definitive concepts for IS (0.17) and PM (0.17), both of which had the highest number of definitive CUIs in the *G_a_* concept sets (403 and 355, respectively).

Precision remained high for both LLMs when evaluated against the full *G_a_* concept sets, exceeding 0.80 for all concepts except CHF for GPT-5 and CHF and PM for Qwen3-32B. Precision was lower when evaluation was restricted to definitive *G_a_* concept set CUIs as all retained CUIs, including context-dependent concepts, were treated as positive predictions.

Overall, these findings demonstrate that LLM-based filtering substantially improved concept set completeness compared with conventional manual curation while maintaining acceptable precision.

### Error analysis and clinical observability of missed concepts

Figure 6 shows that many of the *G_a_* CUIs missed by CUI-Curate (text-embedding-3-large query retrieval plus GPT-5 filtering and classification) were not observed in the sampled MIMIC-III notes, particularly among context-dependent concepts. In contrast, definitive concepts observed in the clinical text were much less likely to be missed, particularly for FO and LVSD concepts.

**Figure 6.**
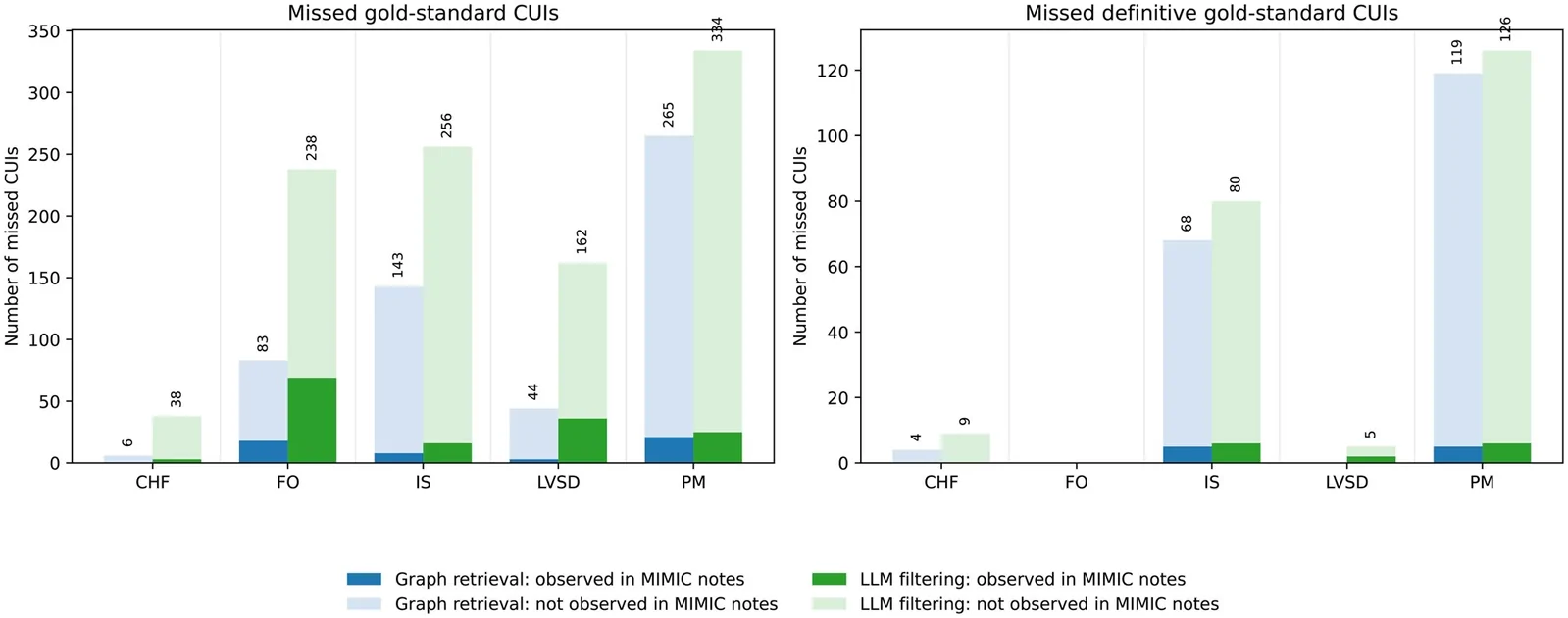
Clinical observability of gold-standard CUIs missed during graph retrieval and filtering stages. Plot shows the number of adjudicated gold-standard CUIs missed at the graph retrieval stage and after the final LLM filtering stage using GPT-5 across five target concepts. Missed CUIs are stratified according to whether they were observed in a sample of 10,000 MIMIC-III clinical notes processed using QuickUMLS. The left panel shows all missed gold-standard CUIs, while the right panel shows only CUIs classified as definitive indicators of the target concept. Abbreviations: CUI, concept unique identifier; CHF, chronic heart failure; FO, fluid overload; IS, ischemic stroke; LVSD, left ventricular systolic dysfunction; PM, poor mobility.

Qualitative review of the concept sets demonstrated CUI-Curate expanded the manually curated concept sets with clinically meaningful terms found in the MIMIC-III notes and absent from the manually collected UMLS browser concept sets (*M*). Examples include *New York Heart Association* functional classes and *cor pulmonale* for CHF; *generalized edema* for FO; *chronic systolic heart failure* for LVSD; *watershed* and *subcortical infarct* for ischemic stroke; and functional descriptors such as *unable to stand up* and *unable to transfer from chair to bed* for PM. CUI-Curate also captured numerous systematic terminology variants, including anatomically specific stroke subtypes (e.g, infarction due to occlusion of individual cerebral arteries) and detailed mobility descriptors (e.g, *difficulty walking up stairs*, *walking on slopes*, and *transferring*).

Residual omissions were concentrated among a small number of interpretable categories. Some reflected prompt-specific decisions, such as exclusion of acute heart failure terms after instructions intended to exclude transient acute cardiac failure. Others involved generic but clinically important descriptors lacking explicit phenotype specificity, anatomically specific vascular terms not explicitly represented in the retrieval query (eg, *vertebral and basilar artery occlusion*), and large families of closely related ontology variants, particularly among physiotherapy and occupational therapy terms for poor mobility. In addition, several missed poor mobility concepts represented neurological conditions commonly associated with impaired mobility, such as *motor neuron disease, cerebral palsy*, and *multiple sclerosis*, where mobility limitation is frequent but not explicitly asserted. Together, these findings suggest that remaining omissions were largely attributable to predictable semantic and ontological complexities rather than systematic failure to identify core phenotype-defining concepts. Full lists of the gold concept sets and their presence in the manual, retrieval, filtered and MIMIC sets are provided in the Supplementary materials.

### Compute vs human resources

One-time UMLS embedding with text-embedding-3-large cost US$8.20 and required just over 2 hours (Table 4). Graph retrieval was fast and incurred negligible cost. GPT-5 filtering and classification cost approximately $0.88 USD per query and required about 21 minutes, whereas Qwen3-32B completed locally in approximately 14 minutes with no API costs beyond query embedding. Manual UMLS browser collection typically required 1–2 hours of clinician time per concept.

**Table 4.** Cost and runtime for CUI-Curate pipeline setup and execution.

Pipeline stage	Method	Approximate cost (USD)	Approximate runtime (minutes)
**One-off infrastructure setup**			
Embedding generation	SapBERT	$0 (run locally)	16.7 min
	e5-large-v2	$0 (run locally)	109.5 min
	Text-embedding-3-large	$8.20	141.2 min
FAISS index creation	SapBERT	$0 (run locally)	2.7 min
	e5-large-v2	$0 (run locally)	4.9 min
	Text-embedding-3-large	$0 (run locally)	7.6 min
**Per-query pipeline execution**			
Graph retrieval	Text-embedding-3-large query embedding + FAISS retrieval	<$0.01	0.14 min
LLM filtering	GPT-5	$0.65	16.29 min
	Qwen3-32B	$0 (run locally)	7.96 min
LLM classification	GPT-5	$0.23	5.02 min
	Qwen3-32B	$0 (run locally)	5.96 min

Note: Embedding generation and FAISS index creation were one-off preprocessing steps performed on the complete UMLS graph. API costs were estimated using the published OpenAI API pricing and the collected input and output token count at each stage (price at the time of execution: text-embedding-3-large input $0.13/million tokens; GPT-5 input $1.25/million tokens, and output $10.00/million tokens). Embedding generation and indexing were performed on a Google Colab L4 GPU instance and graph retrieval, and GPT-5 CUI filtering and classification were performed using a Google Colab HighRAM CPU instance. Qwen3-32B filtering and classification were performed using a Google Colab A100 GPU instance. Google Colab instances were accessed using a Colab Pro account (additional details provided in the Supplementary materials).

## Discussion

This study demonstrates that combining graph-based retrieval over the UMLS with LLM-guided filtering provides a scalable and reproducible framework for clinical concept set curation. Across five lexically heterogeneous target concepts, CUI-Curate generated significantly larger and more comprehensive concept sets than manual UMLS browser searches, which captured only a small proportion of clinically relevant concepts. A single retrieval configuration using text-embedding-3-large achieved high recall for definitive concepts with manageable candidate sets. GPT-5 consistently outperformed manual curation and preserved nearly all definitive phenotype-defining concepts, while open-source Qwen3-32B achieved comparable, though slightly lower, performance. Many missed concepts were not observed in clinical notes, and computational costs were modest relative to the clinician time required for manual concept set development.

### Graph retrieval

Retrieval performance varied substantially across target concepts and was driven primarily by the semantic and ontological characteristics of each rather than by instability of the retrieval method. *CHF* and *LVSD* were retrieved with high recall, reflecting their relatively coherent representation within the UMLS and the presence of well-established terminology. In contrast, FO and PM were more challenging because they are expressed through diverse findings, functional descriptions, and associated conditions rather than a single clearly bounded diagnosis, consistent with previous studies on these phenotypes.^24^^,^^25^

Among the embedding models evaluated, text-embedding-3-large provided the strongest retrieval performance, suggesting that large general-purpose embeddings may outperform domain-specific biomedical models for ontology retrieval tasks. Semantic retrieval alone and unrestricted recursive child expansion rapidly became computationally impractical. CUI-Curate’s combination of semantic retrieval, one-hop expansion, and restricted neighbor limits achieved high recall for definitive concepts while maintaining manageable candidate sets for downstream filtering and classification.

For several concepts, particularly FO, PM, and LVSD, the adjudicated gold-standard sets were intentionally broad and included many context-dependent concepts. This reflected the absence of a universally accepted boundary for these phenotypes, supported by lower IAA for broader phenotypes such as FO and PM compared with more clearly defined concepts like IS. The gold-standard sets were therefore designed to test CUI-Curate’s ability to retrieve a wide range of clinically plausible indicators that can be manually reviewed and tailored to specific downstream applications.

### LLM filtering, clinical observability, and practical utility

LLM-based filtering substantially improved concept set completeness compared with manual UMLS browsing while reducing the candidate sets to a more manageable number of relevant CUIs. GPT-5 achieved higher recall than manual concept sets for all five target concepts and preserved at least 95% of definitive gold-standard CUIs, indicating that the most clinically specific concepts were retained with high reliability. Qwen3-32B consistently outperformed manual curation and approached GPT-5 performance, demonstrating that effective concept set generation is feasible using open-source models.

Many of the concepts missed by CUI-Curate were unlikely to have a substantial impact on downstream clinical text mining. In 10,000 sampled MIMIC-III notes, a large proportion of missed gold-standard CUIs, particularly context-dependent concepts, were not observed in the clinical text, whereas commonly documented definitive concepts were rarely missed. These findings suggest that conventional recall metrics may overstate the practical importance of some omissions.

Qualitative review showed that CUI-Curate captured numerous clinically meaningful and text-observable concepts absent from manual concept sets. Remaining omissions were largely attributable to predictable and interpretable factors, including prompt-specific exclusions, generic descriptors lacking explicit phenotype specificity, incomplete query coverage, and large families of closely related ontology variants. These errors appear amenable to refinement through targeted prompt engineering and iterative review.

The computational requirements of CUI-Curate were modest relative to the clinician effort typically required for manual concept set development. With a one-time UMLS embedding cost of less than US$10 and GPT-5 processing cost of less than US$1 per concept, CUI-Curate offers a cost-efficient approach to automating concept set curation. These low costs enable users to iteratively refine prompts and rerun the pipeline to generate concept sets tailored to specific study objectives.

### Limitations

Several limitations should be considered. First, the evaluation was restricted to five target concepts. Although these were intentionally selected to represent lexically heterogeneous phenotypes and concepts with extensive subtypes, performance for other concepts such as procedures, medications, laboratory findings, and rare diseases remains to be established.

Second, despite the use of multiple embedding models, graph expansion strategies, and pooled relevance assessment, some relevant CUIs may remain unretrieved. Pooled relevance assessment provides a practical and rigorous approach to benchmark construction in a search space exceeding 900,000 UMLS concepts but cannot guarantee absolute completeness without exhaustive review.

Third, for concepts such as FO, PM, and LVSD, the adjudicated gold-standard sets were intentionally broad and included many context-dependent concepts. This reflects the absence of universally accepted phenotype boundaries and the fact that the concept sets are often tailored to specific analytic objectives. Fourth, the distinction between definitive and context-dependent concepts is partly subjective despite formal adjudication. Fifth, clinical observability was assessed in a single corpus (MIMIC-III) using QuickUMLS and may not generalize to other healthcare settings or named entity recognition tools.

### Future work

Future work could extend the evaluation to a broader range of concept types, particularly less standardized domains such as nursing and rehabilitation terminologies. CUI-Curate is best viewed as an iterative concept design tool rather than a fully automated one-step automated solution. Because graph retrieval is rapid and filtering costs are modest, users can inspect retrieved concepts, refine target descriptions and prompts and rerun the pipeline to generate study-specific concept sets tailored to different analytical objectives and balances between sensitivity and specificity. This process may be further improved by incorporating richer concept descriptions, including clinical diagnostic criteria and relevant clinical literature. Additional engineering work may reduce runtime through parallel processing and optimized prompting. Finally, downstream evaluations in concept normalization, cohort definition, and automated registry creation will be important to establish the clinical and research value of CUI-Curate-generated concept sets, which will be explored in future studies.

## Conclusion

CUI-Curate is a scalable and reproducible framework for automated UMLS concept set curation that combines graph-based retrieval with LLM-guided filtering and classification. Across five clinical concepts, it consistently outperformed manual UMLS browser searches, generating larger and more complete concept sets while maintaining acceptable precision.

The findings show that concept set curation can benefit from a GraphRAG framework in which graph-based retrieval constrains the search space, and LLMs prioritize clinically relevant concepts for expert review. By providing a practical and cost-efficient approach to generating clinician-reviewable, study-specific concept sets, CUI-Curate supports more consistent and maintainable use of UMLS-based concept normalization in clinical NLP and phenotyping applications.

## Supplementary Material

ocag110_Supplementary_Data

## Data Availability

The CUI-Curate framework is available at https://github.com/vickyblake/CUICurate. The gold-standard concept sets created by this project are provided in the Supplementary materials. Unified Medical Language System data tables are distributed by the U.S. National Library of Medicine under license and are not publicly shareable. Researchers can apply for a free UMLS Metathesaurus license directly through the National Library of Medicine at https://www.nlm.nih.gov/research/umls/index.html. MIMIC-III data can be accessed through PhysioNet here: https://physionet.org/content/mimiciii/1.4/

## References

[1] Xu H , Demner Fushman D, Hong N, Raja K. Medical concept normalization In: Xu H, Demner Fushman D, editors. Natural Language Processing in Biomedicine: A Practical Guide. Cham: Springer International Publishing; 2024:137-64.

[2] Jing X. The unified medical language system at 30 years and how it is used and published: systematic review and content analysis. JMIR Med Inform. 2021;9:e20675. 10.2196/20675 PMC8433943 34236337

[3] Gold S , Batch A, McClure R, Jiang G, Kharrazi H, Saripalle R, Huser V, Weng C, Roderer N, Szarfman A, Elmqvist N, Gotz D. Clinical concept value sets and interoperability in health data analytics. AMIA Annu Symp Proc. 2018;2018:480-489. PMC6371254 30815088

[4] Soldaini L , Goharian N. Quickumls: a fast, unsupervised approach for medical concept extraction. MedIR workshop, sigir; 2016. 1-4.

[5] Kraljevic Z , Bean D, Mascio A, Roguski L, Folarin A, Roberts A, Bendayan R, Dobson R. MedCAT—medical concept annotation tool. arXiv pre-print server arXiv: 191210166, 2019, preprint: not peer reviewed. 10.48550/arXiv.1912.10166

[6] Rodriguez VA , Tony S, Thangaraj P, Pang C, Kalluri KS, Jiang X, Ostropolets A, RuiJun C, Karthik N, Ryan P. Phenotype concept set construction from concept pair likelihoods. AMIA Annu Symp Proc. 2020;2020:1080-1089. PMC8075469 33936484

[7] UMLS Metathesaurus Browser. Accessed September 28, 2025. https://uts.nlm.nih.gov/uts/umls

[8] Athena Search Terms. Accessed September 28, 2025. https://athena.ohdsi.org/search-terms/start

[9] Kipp M. From GPT-3.5 to GPT-4.o: a leap in AI’s medical exam performance. Information. 2024;15:543.

[10] Lewis P , Perez E, Piktus A, Petroni F, Karpukhin V, Goyal N, Kuttler H, Lewis M, Yih W, Rocktaschel T, Riedel S, Kiela D. Retrieval-augmented generation for knowledge-intensive NLP tasks. In: *Proceedings of the 34th International Conference on Neural Information Processing Systems*. Vancouver, BC, Canada: Curran Associates Inc. 2020. https://proceedings.neurips.cc/paper_files/paper/2020/file/6b493230205f780e1bc26945df7481e5-Paper.pdf

[11] Han H , Wang Y, Shomer H, Guo K, Ding J, Lei Y, Halappanavar M, Rossie RA, Mukherjee S, Tang X, He Q, Hua Z, Long B, Zhao T, Shah N, Javari A, Xia Y, Tang J. Retrieval-augmented generation with graphs (GraphRAG). arXiv preprint arXiv: 250100309, 2024, preprint: not peer reviewed. 10.48550/arXiv.2501.00309

[12] Lukyanchikov N , Kawamoto K. Evaluation of discrepancies among national library of medicine (NLM) value set authority center (VSAC) ICD-10-CM value sets: case study for diagnoses of common chronic conditions, implications, and potential solutions. AMIA Annu Symp Proc. 2023;2023:1087-1095. PMC10785892 38222435

[13] Dobbins NJ. Generalizable and scalable multistage biomedical concept normalization leveraging large language models. Res Synth Methods. 2025;16:479-490. 10.1017/rsm.2025.9 PMC12527512 41626935

[14] Banf M , Kuhn J. Tripartite-GraphRAG via Plugin Ontologies. arXiv preprint arXiv: 250419667v3, 2025, preprint: not peer reviewed. 10.48550/arXiv.2504.19667

[15] Kim D, , Yoo S, , Jeong O. MedSumGraph: enhancing GraphRAG for medical QA with summarization and optimized prompts. Artif Intell Med. 2026;172:103311. 10.1016/j.artmed.2025.10331141319399 41319399

[16] Goel S , Lee RJ, Ramchandran K. SAGE: A realistic benchmark for semantic understanding. arXiv preprint arXiv: 2509213102025, preprint: not peer reviewed.

[17] Liu F , Shareghi E, Meng Z, Basaldella M, Collier N. Self-alignment pretraining for biomedical entity representations. In *Proceedings of the 2021 conference of the North American chapter of the association for computational linguistics: Human language technologies*. Association for Computational Linguistics; 2021:4228-4238. 10.18653/v1/2021.naacl-main.334

[18] Excoffier J-B , Roehr T, Figueroa A, Papaioannou J-M, Bressem K, Ortala M. Generalist embedding models are better at short-context clinical semantic search than specialized embedding models. arXiv preprint arXiv: 240101943, 2024, preprint: not peer reviewed.

[19] Wang L , Yang N, Huang X, Jiao B, Yang L, Jiang D, Majumder R, Wei F. Text embeddings by weakly-supervised contrastive pre-training. arXiv preprint arXiv: 221203533, 2022, preprint: not peer reviewed.

[20] Douze M , Guzhva A, Deng C, et al The Faiss Library. IEEE Trans Big Data. 2026;12 2:346-361. 10.1109/TBDATA.2025.3618474

[21] Yang A , Li A, Yang B, et al Qwen3 technical report. arXiv preprint arXiv: 250509388, 2025, preprint: not peer reviewed.

[22] Sanderson M. Test collection based evaluation of information retrieval systems. Found Trends Inf Retr. 2010;4:247-375.

[23] Voorhees EM , Soboroff I, Lin J. Can old TREC collections reliably evaluate modern neural retrieval models? arXiv preprint arXiv: 220111086, 2022, preprint: not peer reviewed.

[24] Thieu T , Maldonado JC, Ho P-S, et al A comprehensive study of mobility functioning information in clinical notes: entity hierarchy, corpus annotation, and sequence labeling. Int J Med Inform. 2021;147:104351. 10.1016/j.ijmedinf.2020.104351 PMC8104034 33401169

[25] Messmer AS , Moser M, Zuercher P, Schefold JC, Müller M, Pfortmueller CA. Fluid overload phenotypes in critical illness—a machine learning approach. J Clin Med. 2022;11:336. 10.3390/jcm11020336 PMC8780174 35054030

