# Supplementary material for: CUI-Curate: a GraphRAG-based framework for automated clinical concept curation for NLP applications
Source: J Am Med Inform Assoc. 2026 Jun 26;33(10):1898–909. doi: 10.1093/jamia/ocag110 (PMC13630293; doi:10.1093/jamia/ocag110)
Supplement: Supplementary file 1 [file ocag110_supplementary_data.zip › CUICurate_Supplementary_JAMIA_11.05.2026_clean.docx]

Supplementary Material

Table of Contents

[Computing environment	2](#_Toc229432606)

[Manual and Gold-Standard Concept Set Agreement Statistics	2](#_Toc229432607)

[Supplementary Table 1. Overlap between manually collected UMLS browser concept sets (M).	2](#_Toc229432608)

[Supplementary Table 2. Inter-annotator agreement for gold-standard (Ga) concept sets	3](#_Toc229432609)

[Graph Retrieval Settings and Additional Experiments	4](#_Toc229432610)

[Supplementary Table 3. Definitions of graph retrieval settings used to limit the graph retrieval of candidate CUIs to control prompt noise and length	4](#_Toc229432611)

[Target Concept Query Optimisation	5](#_Toc229432612)

[Target Concept Description Queries	6](#_Toc229432613)

[Supplementary Table 5. Target concept description text used as queries for CUI retrieval, semantic types and additional LLM filtering instrictions and classification few-shots.	6](#_Toc229432614)

[LLM Prompting	14](#_Toc229432615)

[LLM Filtering Prompt Template	14](#_Toc229432616)

[LLM Classification Prompt Template	15](#_Toc229432617)

[Graph Retrieval Performance	17](#_Toc229432618)

[LLM Filtering Performance	19](#_Toc229432619)

[Supplementary Table 7. LLM filtering performance.	19](#_Toc229432620)

[Supplementary Figure 6. Supplementary Figure 6. LLM total run time and cost across models and CUI chunk sizes for chronic heart failure concept (350 CUIs processed in total)	21](#_Toc229432621)

# Computing environment

Google Colab with a Google Colab Pro account was used for all framework development and testing . The graphRAG components (graph, embeddings and indexes) were built using an L4 GPU instance type, the GPT-5 experiments were run using a high RAM CPU instance, accessing the model through the OpenAI API. The Qwen3-32B experiments were run using an A100 high RAM GPU instance.

# Manual and Gold-Standard Concept Set Agreement Statistics

The manually collected concept sets ***(M)*** showed low overlap between the annotators (Jaccard = 0.08-0.37; overlap coefficient = 0.19-0.65) due to the very large CUI search space and differing search paths within the UMLS Metathesaurus browser. Agreement between the two annotators for the gold-standard (*G_a_*) manually adjudicated concept sets was generally high for inclusion decisions (percent agreement = 83.9-92.6%; _K_ = 0.64-0.69) across concepts. Category agreement CUIs was more variable (percent agreement 67.5-94.1%; _K_ = 0.32-0.84).

## Supplementary Table 1. Overlap between manually collected UMLS browser concept sets (M).

|  | Total CUIs | Intersection Size | Jaccard | Overlap CoeFfICIENT |
| --- | --- | --- | --- | --- |
| Fluid Overload | 41 | 15 | 0.37 | 0.65 |
| Ischaemic Stroke | 166 | 24 | 0.15 | 0.27 |
| LV Systolic Dysfunction | 68 | 12 | 0.18 | 0.31 |
| Poor Mobility | 148 | 12 | 0.08 | 0.19 |

## Supplementary Table 2. Inter-annotator agreement for gold-standard (Ga) concept sets

| Metric | Chronic heart failure | Fluid overload | Ischaemic stroke | LVSD | Poor Mobility |
| --- | --- | --- | --- | --- | --- |
| Total CUIs in pooled superset (n) | 1835 | 2071 | 1662 | 2111 | 2140 |
| Annotator 1 included (n, %) | 161 (8.8%) | 292 (14.1%) | 674 (40.6%) | 264 (12.5%) | 779 (36.4%) |
| Annotator 2 included (n, %) | 410 (22.3%) | 205 (9.9%) | 619 (37.2%) | 291 (13.8%) | 512 (23.9%) |
| Include Agreement (%) | 82.3% | 90.2% | 93.9% | 94.5% | 73.7% |
| Include Cohen’s Kappa | 0.349 | 0.542 | 0.872 | 0.757 | 0.387 |
| Inclusion conflicts (n) | 325 | 202 | 101 | 117 | 563 |
| Category Agreement (%) | 67.5% | 82.4% | 85.7% | 75.3% | 73.6% |
| Category Cohen’s Kappa | 0.306 | 0.519 | 0.664 | 0.467 | 0.431 |
| Category conflicts (n) | 40 | 26 | 85 | 54 | 96 |
| Final included CUIs (n, %) | 163 (8.9%) | 321 (15.5%) | 675 (40.6%) | 287 (13.6%) | 655 (30.6%) |
| Final definitive CUIs (n, %) | 114 (69.9%) | 23 (7.2%) | 403 (59.7%) | 51 (17.8%) | 355 (54.2%) |
| Final context-dependent CUIs (n, %) | 49 (30.1%) | 298 (92.8%) | 273 (40.4%) | 236 (82.2%) | 300 (45.8%) |

# Graph Retrieval Settings and Additional Experiments

## Supplementary Table 3. Definitions of graph retrieval settings used to limit the graph retrieval of candidate CUIs to control prompt noise and length

| **Retrieval Setting** | **Description** |
| --- | --- |
| Target CUI | The CUI with the top semantic similarity to the target concept description |
| K | Top-*k* most similar CUIs as initial seeds |
| Children | Descendent nodes of the seed CUIs |
| Hops | Number of edge traversals from the seed CUIs (e.g. synonyms and broader terms that are not descendants of K) |
| Max Neighbours | Maximum threshold number of nearest neighbour CUIs retrieved from the neighbourhood of the target CUI (limited prompt length and reduced noise) |
| Semantic Type | UMLS semantic types eligible for retrieval |

Supplementary Table 4. Semantic retrieval only strategy at various bands of target recall for the five target concepts using the adjudicated gold-standard concept sets. The number of CUIs retrieved in the candidate concept sets quickly escalates for higher target recall values, particularly for fluid overload (FO) and poor mobility (PM).

|  | **CHF** | | **FO** | | **LVSD** | | **IS** | | **PM** | |
| --- | --- | --- | --- | --- | --- | --- | --- | --- | --- | --- |
| **Target recall** | **CUIs retrieved** | **Gold missed** | **CUIs retrieved** | **Gold missed** | **CUIs retrieved** | **Gold missed** | **CUIs retrieved** | **Gold missed** | **CUIs retrieved** | **Gold missed** |
| 0.60 | 138 | 64 | 591 | 134 | 328 | 99 | 422 | 235 | 499 | 176 |
| 0.70 | 179 | 48 | 812 | 101 | 411 | 74 | 518 | 176 | 695 | 132 |
| 0.80 | 272 | 32 | 1179 | 67 | 573 | 49 | 646 | 117 | 1084 | 88 |
| 0.85 | 329 | 24 | 2029 | 50 | 718 | 37 | 708 | 88 | 1805 | 66 |
| 0.90 | 382 | 16 | 4810 | 33 | 921 | 24 | 848 | 58 | 3060 | 44 |
| 0.95 | 505 | 8 | 12453 | 16 | 1398 | 12 | 1110 | 29 | 7220 | 22 |
| 1.00 | 763 | 0 | 60426 | 0 | 4022 | 0 | 5006 | 0 | 32967 | 0 |

# Target Concept Query Optimisation

To optimise the recall of the retrieval query for each target concept, GPT-5-mini was used to craft descriptions of the target concept using the below prompt. The 3 LLM-generated descriptions were then reviewed by a clinician and combined to form one description that covered the key synonyms, subtypes and variant clinical phrases that would indicate the target concept.

```
You are a clinical NLP expert optimising retrieval recall for UMLS GraphRAG.
 Target concept: {concept}

 Write 3 alternative, recall-oriented descriptions (<=120 words each) to use as dense retrieval queries.
 Each description MUST:
      - Include synonyms and lexical variants (incl. US/UK spellings)
      - Include common abbreviations if applicable
      - Mention key indicators (specific signs, symptoms, measurements, labs, procedures, and subtypes)
      - Avoid broad, non-specific symptoms
      - Be one paragraph per variant
```

# Target Concept Description Queries

Supplementary Table 5. Target concept description text used as queries for CUI retrieval, semantic types and additional LLM filtering instrictions and classification few-shots. Concept descriptions embedded and used to calculate cosine similarity to UMLS graph nodes and guide LLM prompt filtering and classification of CUIs

| **Target Concept** | **Concept Description Query Embedded and** | **Semantic Types** | **LLM filtering instructions** | **LLM classification few-shots** |
| --- | --- | --- | --- | --- |
| Chronic heart failure | Synonyms, equivalent terms and abbreviations:  Chronic heart failure.  Chronic cardiac failure,  congestive heart failure,  chronic congestive cardiac failure,  chronic cardiac insufficiency,  pump failure  Subtypes and variants:  Heart failure with reduced ejection fraction (HFrEF)  Heart failure with preserved ejection fraction (HFpEF)  Heart failure with mid-range ejection fraction (HFmrEF)  systolic heart failure  diastolic heart failure,  right‑sided heart failure,  left‑sided heart failure,  bilventricular heart failure.  Objective indicators:  Ventricular dysfunction.  Reduced LVEF on echocardiography  Cardiomyopathy  Exertional dyspnoea / dyspnea  Orthopnoea / orthopnea  Paroxysmal nocturnal dyspnoea / dyspnea  Elevated BNP or NT‑proBNP  Cardiomegaly or pulmonary congestion on chest X‑ray  New York Heart Association score (NYHA) | Disease or Syndrome,  Pathologic Function,  Diagnostic Procedure,  Health Care Activity,  Finding,  Laboratory or Test Result | Include:  - "heart failure" with unspecified acuity, “acute‑on‑chronic” presentations and compensated or decompensated states.  - concepts for heart failure hospitalisations or care events.  Exclude:  - concepts related to acute cardiac failure (e.g. cardiogenic shock) that are are likely acute issues that may resolve, for example sepsis, post-procedure.  - concepts related to fetal or neonate cardiac failure. | definitive: chronic congestive cardiac failure, heart failure with reduced ejection fraction  context dependent: heart failure, congestive cardiac failure |
| Fluid overload | Synonyms, equivalent terms and abbreviations:  Systematic fluid overload  Systemic volume overload,  hypervolemia,  fluid retention,  fluid excess,  circulatory overload,  volume expansion.  volume overload  Subtypes and variants:  Pulmonary edema/ pulmonary oedema  Dependent (peripheral) edema  Anasarca (generalised edema)  Transfusion-associated circulatory overload (TACO)  Iatrogenic volume overload  Renal failure-associated volume overload  Cardiogenic volume overload.  Objective indicators:  Positive fluid balance  acute weight gain,  elevated central venous pressure (CVP),  Jugular venous distention (elevated JVP)  Generalised oedema  Generalised edema  dilated non‑collapsible Inferior Vena Cava,  Vascular congestion  elevated B-type natriuretic peptide (BNP)  elevated N-terminal pro-B-type natriuretic peptide (NT‑proBNP),  Kerley B lines,  Interstitial edema  Alveolar edema  Pleural effusion,  requirement for dialysis  requirement for ultrafiltration,  response to loop diuretics  response to ultrafiltration,  Bilateral peripheral edema  Bilateral pitting edema,  Ascites | Disease or Syndrome,  Pathologic Function,  Diagnostic Procedure,  Health Care Activity,  Finding,  Laboratory or Test Result,  Sign or Symptom | Include:  - concepts for hospitalisations or care events related to fluid overload.  Exclude:  - concepts related to fetal fluid overload. | definitive: pulmonary oedema due to fluid overload, hypervolaemia  context dependent: oedema of the lower limbs, fluid imbalance |
| Left ventricular systolic dysfunction | Synonyms, equivalent terms and abbreviations:  LV systolic dysfunction,  Left ventricular dysfunction  Left ventricular systolic failure,  systolic heart failure,  heart failure with reduced ejection fraction, HFrEF,  impaired left ventricular contractility  acute systolic dysfunction  chronic systolic dysfunction  Subtypes and variants:  Dilated cardiomyopathy  post‑MI/ischaemic (ischemic) cardiomyopathy  Post-MI cardiomyopathy  Ischaemic cardiomyopathy  non‑ischaemic cardiomyopathy,  myocarditis‑related cardiomyopathy,  anthracycline induced cardiomyopathy  toxin‑induced cardiomyopathy  post‑infarct remodelling,  cardiomegaly.  Objective indicators:  low stroke volume  cardiac output,  depressed LVEF/EF (eg EF <40% or <35%),  reduced ejection fraction  reduced fractional shortening,  global hypokinesis  regional wall‑motion abnormality  abnormal transthoracic/TEE echocardiography,  pulmonary oedema/edema,  raised pulmonary capillary wedge pressure,  low cardiac index (<2.2 L/min/m2),  elevated BNP  elevated NT‑proBNP,  S3 gallop | Disease or Syndrome,  Pathologic Function,  Diagnostic Procedure,  Health Care Activity,  Finding,  Laboratory or Test Result, | Include:  - concepts for hospitalisations or care events related to Left Ventricular Systolic Dysfunction.  Exclude:  - concepts related to foetal ventricular function.  - concepts related to clinical manifestations of systolic dysfunction like pulmonary oedema without the concept specifying that it is related to LV dysfunction. | definitive: left‑sided systolic failure, heart failure with reduced ejection fraction  context dependent: dilated cardiomyopathy, ventricle wall‑motion abnormality |
| Ischaemic Stroke | Synonyms, equivalent terms and abbreviations:  stroke,  ischemic stroke  cerebrovascular accident (CVA),  cerebral infarction,  acute brain infarct,  brain ischemia.  Brain ischaemia  Subtypes and variants:  Thromboembolic stroke  thrombotic infarct,  embolic infarct,  cardioembolic infarct  lacunar infarct,  small-vessel infarct  watershed infarct  large vessel occlusion (LVO) infarct.  Large artery atherosclerotic stroke (TOAST)  Middle Cerebral Artery infarct  Internal Carotid Artery territory infarct,  Anterior Cerebral Artery infarct,  Posterior Cerebral Artery infarct,  basal ganglia infarct,  brainstem infarct,  cerebellum infarct.  Cryptogenic stroke  Objective indicators:  sudden focal deficits,  unilateral weakness  unilateral hemiparesis,  sensory lacunar syndromes,  cortical signs,  facial droop,  sudden aphasia (expressive/global),  expressive aphasia  global aphasia  dysarthria,  neglect,  homonymous hemianopia,  ataxia  brainstem signs  diplopia,  dysphagia  National Institute of Health Stroke score (NIHSS).  CTB hypodensity,  CTB perfusion mismatch,  CTB occlusion  MRA brain occlusion  Carotid stenosis  DWI MRI diffusion restriction,  Intravenous thrombolysis.  mechanical thrombectomy (MT),  endovascular thrombectomy (EVT). | Disease or Syndrome,  Pathologic Function,  Diagnostic Procedure,  Health Care Activity,  Finding,  Laboratory or Test Result, | Include:  - concepts for hospitalisations or care events related to ischaemic stroke.  Exclude:  - concepts related to foetal. | definitive: cerebral infarction, MCA/ICA territory infarct  context dependent: acute cerebrovascular accident (CVA), stroke |
| Poor Mobility | Synonyms, equivalent terms and abbreviations:  poor mobilization/mobilisation;  reduced mobility;  decreased mobility  difficult ambulation  impaired mobility  limited mobility  mobility limitation  non‑ambulatory;  difficulty ambulating  immobilised/immobilized  subtypes and variants:  Gait disturbance / Gait disorder / Impaired gait  Balance impairment / postural instability  Reduced independent ambulation  Ataxic gait.  Wheelchair‑dependent mobility  Difficulty walking.  Difficulty ambulating.  Does not walk.  Does not mobilise.  Transfer impairment.  Sit-to-stand impairment.  Wheelchair-bound.  Bed-bound.  Objective indicators:  Transfer dependence.  Assistance-dependent mobilisation.  Uses walking stick.  Uses cane.  Uses walker.  Uses rollator.  Uses wheelchair.  Slow gait speed.  Abnormal tandem or unsteady gait  Timed Up and Go test > 12 seconds.  Reduced six-minute walk test distance (6MWT).  Reduced 10-metre walk performance.  Inability to perform five times sit-to-stand (STS).  Recurrent falls. | Disease or Syndrome,  Pathologic Function,  Diagnostic Procedure,  Health Care Activity,  Finding,  Laboratory or Test Result, | Include:  - concepts for hospitalisations or care events related to Poor Mobility.  - concepts related to use of mobility aids.    Exclude:  - concepts related to the measurement of mobility without any assertion that the mobility is limited or poor.  - concepts that are Conditions or impairments associated with poor mobility where mobility impairment is not explicit or inevitable (e.g. early stages of Parkinson's disease) | definitive: difficulty walking, use of walking stick, use of wheelchair  context dependent: recurrent falls, ataxia, difficulty kneeling, decreased range of movement in lower body region |

# LLM Prompting

## LLM Filtering Prompt Template

```
You are a biomedical assistant working with a UMLS-based knowledge graph.

Task: Filter the CUIs provided below that should reasonably be considered to indicate the target concept.

Include if:
    1) It directly denotes the target concept or is a clinically equivalent synonym.
    2) It is a subtype/child that is commonly subsumed by the target concept without additional qualifiers.
    3) It is a closely bound variant naming (spelling variants, common aliases).
    4) It is a procedure, therapy, medication, lab/test, measurement, risk factor, aetiology, complication,
       manifestation, generic finding, or care event (e.g., specialty clinic attendance, disease-management programs, surgery admissions) that is widely recognised as a proxy or near-unique indicator of the target concept.

Exclude if:
    1) It is a generic parent/container concept that is not commonly used as the disease name.
    2) It expresses only suspicion, family history, or negation of the target concept.
    3) It is clearly unrelated or ambiguous.

Prefer inclusion to preserve recall for direct assertion concepts. For conditional categories in 4),
include only when specificity is high; if uncertain about specificity, exclude.

Target concept: {concept_name} (CUI: {concept_cui})

Target concept description/aliases: {target_description}

Special include/exclude instructions: {special_instructions}

OUTPUT FORMAT (strict):
     - Return ONLY valid JSON with exactly one key: "selected_cuis".
     - "selected_cuis" MUST be an array of CUIs (strings matching ^C\\d{7}$).
     - You may ONLY output CUIs from the provided candidate list below.
     - No prose, no comments, no extra keys.
     - De-duplicate and sort CUIs ascending for stability.

Candidates:
        {cui}: {cui_name}
        {cui}: {cui_name}
        {cui}: {cui_name}
```

## LLM Classification Prompt Template

```
You are a biomedical assistant working with UMLS CUIs.

Task: Classify every provided CUI listed below into exactly one of the categories as defined below.

Rules:
    1) "definitive": direct, unambiguous assertion of the target concept (or strict synonyms).
    2) "context_dependent": modifiers, tests, measurements, risk factors, causes, complications, manifestations: include parents only if the term commonly used to refer to the target concept; subtypes that need qualifiers.
    3) Tie-breakers: when uncertain, prefer "context_dependent"; choose specific assertion over generic parent.
    4) No omissions, no duplicates.

Target concept: {target_description}

Few-shot examples: {fewshots}

Return ONLY valid JSON with exactly these keys: "definitive" and "context_dependent".
Values MUST be arrays of CUIs (strings like "C1234567") drawn ONLY from the provided list.
Do NOT include names, comments, or extra keys.
   Example:
    	{
      		"definitive": ["C0000001", "C0000002"],
      		"context_dependent": ["C0000003"]
    	}

CUIs:
	{cui}: {cui_name}
	{cui}: {cui_name}
	{cui}: {cui_name}
```

# Graph Retrieval Performance

## Evaluation Framework

To evaluate the automated system, each stage of the pipeline was assessed separately. All evaluations were conducted on the CUIs accessible to each method (i.e., the method’s retrieval or inclusion space) to ensure fair comparisons.

1. **Graph Retrieval Performance**

Graph retrieval (***R***) performance was evaluated against the clinician benchmark set ***M.*** Recall was the primary optimisation target and was calculated as:

$${Recall}_{retrieval}= \frac{|M \cap R|}{|M|}$$

2. **Filtering Performance**

The LLM filtering step and the manual benchmark were evaluated against the adjudicated gold-standard ***Gₐ***, which comprises the pooled CUIs retrieved from three embedding models. These CUIs were independently reviewed and annotated by clinicians with consensus adjudication.

**Evaluation of LLMs**

LLM predictions (***P****ˡˡᵐ)* were compared with the gold-standard ***Gₐ***. Evaluation was restricted to CUIs retrievable under GraphRAG (i.e., ***Gₐ ∩ R***), matching the LLMs’ search space.

$$\text{Recall}_{\text{llm}}=\frac{\mid G_{a}\cap P^{llm}\mid}{\mid G_{a}\cap R\mid}$$

$$\text{Precision}_{\text{l}\text{lm}}=\frac{\mid G_{a}\cap P^{llm}\mid}{\mid P^{llm}\mid}$$

**Evaluation of the Manual Benchmark**

Because ***Gₐ*** is constrained by GraphRAG retrieval, it may omit valid CUIs collected by clinicians simply because retrieval did not surface them. Treating such CUIs as false positives would therefore misrepresent manual performance.

To ensure a fair comparison, all CUIs proposed by clinicians in the manual concept sets (***Pᴹ***) were treated as condition-positive for inclusion evaluation. Manual performance therefore focuses on recall of the adjudicated gold:

$$\text{Recall}_{\text{M}}=\frac{\mid G_{a}\cap P^{M}\mid}{\mid G_{a}\mid}$$

3. **Definitive CUI Performance**

Definitive CUI performance was evaluated only for CUIs included during the preceding inclusion stage for each method. Each CUI, was evaluated against with the gold-standard classification in ***Gₐ****,* and precision, recall, and F1-scores were computed.

Supplementary Table 6. Semantic retrieval only results. Number of CUIs retrieved and number of gold-standard CUIs missed at various recall values

|  | CHF | | FO | | LVSD | | IS | | PM | |
| --- | --- | --- | --- | --- | --- | --- | --- | --- | --- | --- |
| Target recall | CUIs retrieved | Gold missed | CUIs retrieved | Gold missed | CUIs retrieved | Gold missed | CUIs retrieved | Gold missed | CUIs retrieved | Gold missed |
| 0.6 | 138 | 64 | 591 | 134 | 328 | 99 | 422 | 235 | 499 | 176 |
| 0.7 | 179 | 48 | 812 | 101 | 411 | 74 | 518 | 176 | 695 | 132 |
| 0.8 | 272 | 32 | 1179 | 67 | 573 | 49 | 646 | 117 | 1084 | 88 |
| 0.85 | 329 | 24 | 2029 | 50 | 718 | 37 | 708 | 88 | 1805 | 66 |
| 0.9 | 382 | 16 | 4810 | 33 | 921 | 24 | 848 | 58 | 3060 | 44 |
| 0.95 | 505 | 8 | 12453 | 16 | 1398 | 12 | 1110 | 29 | 7220 | 22 |
| 1.0 | 763 | 0 | 60426 | 0 | 4022 | 0 | 5006 | 0 | 32967 | 0 |

Supplementary Table 7. Retrieval results at the selected graph retrieval settings for five target concepts.

| **concept** | **model** | **k** | **max_neighbors** | **hops** | **retrieved_n** | **recall** | **precision** | **f1** | **recall_def** |
| --- | --- | --- | --- | --- | --- | --- | --- | --- | --- |
| Chronic Heart Failure | text-embedding-3-large | 500 | 1000 | 1 | 758 | 0.962 | 0.204 | 0.337 | 0.960 |
| Fluid Overload | text-embedding-3-large | 500 | 1000 | 1 | 768 | 0.721 | 0.316 | 0.439 | 0.961 |
| Ischaemic Stroke | text-embedding-3-large | 500 | 1000 | 1 | 764 | 0.857 | 0.660 | 0.746 | 0.900 |
| LV Systolic Dysfunction | text-embedding-3-large | 500 | 1000 | 1 | 743 | 0.895 | 0.298 | 0.448 | 0.925 |
| Poor Mobility | text-embedding-3-large | 500 | 1000 | 1 | 782 | 0.666 | 0.409 | 0.507 | 0.765 |

# LLM Filtering Performance

Supplementary Table 7. LLM filtering performance. Number of CUIs included in each concept set, with mean recall, precision and F1-score across five runs per concept for GPT-5 and Qwen3-32B, and compared with the manual UMLS browser collected concept sets. Each concept set was evaluated against the adjudicated gold-standard CUIs.

| **Evaluation Type** | **Concept** | **Method** | **Gold CUIs, n** | **Predicted CUIs, n** | **Recall** | **Precision** | **F1** |
| --- | --- | --- | --- | --- | --- | --- | --- |
| All CUIs | CHF | Manual | 163 | 98 | 0.60 | 1.00 | 0.75 |
|  |  | GPT-5 | 157 | 193 (2) | 0.82 (0.02) | 0.66 (0.01) | 0.73 (0.01) |
|  |  | Qwen3-32B | 157 | 178 (7) | 0.69 (0.03) | 0.61 (0.00) | 0.65 (0.02) |
|  | FO | Manual | 325 | 30 | 0.09 | 1.00 | 0.17 |
|  |  | GPT-5 | 238 | 86 (4) | 0.33 (0.01) | 0.91 (0.02) | 0.48 (0.02) |
|  |  | Qwen3-32B | 238 | 48 (8) | 0.17 (0.02) | 0.83 (0.06) | 0.28 (0.03) |
|  | IS | Manual | 702 | 130 | 0.19 | 1.00 | 0.31 |
|  |  | GPT-5 | 532 | 423 (6) | 0.78 (0.01) | 0.98 (0.00) | 0.87 (0.01) |
|  |  | Qwen3-32B | 532 | 401 (0) | 0.69 (0.00) | 0.92 (0.00) | 0.79 (0.00) |
|  | LVSD | Manual | 290 | 55 | 0.19 | 1.00 | 0.32 |
|  |  | GPT-5 | 243 | 175 (5) | 0.64 (0.01) | 0.89 (0.02) | 0.74 (0.01) |
|  |  | Qwen3-32B | 243 | 80 (0) | 0.30 (0.00) | 0.91 (0.00) | 0.45 (0.00) |
|  | PM | Manual | 677 | 92 | 0.14 | 1.00 | 0.24 |
|  |  | GPT-5 | 390 | 372 (15) | 0.77 (0.02) | 0.81 (0.02) | 0.79 (0.01) |
|  |  | Qwen3-32B | 390 | 352 (0) | 0.66 (0.00) | 0.73 (0.00) | 0.70 (0.00) |
| Definitive CUIs | CHF | Manual | 114 | 98 | 0.79 | 0.92 | 0.85 |
|  |  | GPT-5 | 110 | 193 (2) | 0.96 (0.01) | 0.55 (0.01) | 0.70 (0.01) |
|  |  | Qwen3-32B | 110 | 178 (7) | 0.76 (0.04) | 0.47 (0.01) | 0.58 (0.02) |
|  | FO | Manual | 23 | 30 | 0.61 | 0.47 | 0.53 |
|  |  | GPT-5 | 23 | 86 (4) | 0.97 (0.04) | 0.26 (0.01) | 0.41 (0.01) |
|  |  | Qwen3-32B | 23 | 48 (8) | 0.77 (0.02) | 0.38 (0.08) | 0.51 (0.06) |
|  | IS | Manual | 403 | 130 | 0.17 | 0.54 | 0.26 |
|  |  | GPT-5 | 335 | 423 (6) | 0.95 (0.01) | 0.75 (0.01) | 0.84 (0.01) |
|  |  | Qwen3-32B | 335 | 401 (0) | 0.80 (0.00) | 0.67 (0.00) | 0.73 (0.00) |
|  | LVSD | Manual | 51 | 55 | 0.55 | 0.51 | 0.53 |
|  |  | GPT-5 | 51 | 175 (5) | 0.95 (0.01) | 0.28 (0.01) | 0.43 (0.01) |
|  |  | Qwen3-32B | 51 | 80 (0) | 0.65 (0.00) | 0.41 (0.00) | 0.50 (0.00) |
|  | PM | Manual | 355 (0) | 92 | 0.17 (0.00) | 0.64 (0.00) | 0.26 (0.00) |
|  |  | GPT-5 | 236 (0) | 372 (15) | 0.95 (0.01) | 0.60 (0.02) | 0.73 (0.02) |
|  |  | Qwen-32B | 236 (0) | 352 (0) | 0.76 (0.00) | 0.51 (0.00) | 0.61 (0.00) |
